# Generation of Myocardial Wall Surface Meshes from Segmented MRI

**DOI:** 10.1155/2009/313517

**Published:** 2009-12-08

**Authors:** Oskar Škrinjar, Arnaud Bistoquet

**Affiliations:** ^1^Department of Biomedical Engineering, Georgia Institute of Technology, Atlanta, GA 30332, USA; ^2^Department of Electrical and Computer Engineering, Georgia Institute of Technology, Atlanta, GA 30332, USA

## Abstract

This paper presents a novel method for the generation of myocardial wall surface meshes from segmented 3D MR images, which typically have strongly anisotropic voxels. The method maps a premeshed sphere to the surface of the segmented object. The mapping is defined by the gradient field of the solution of the Laplace equation between the sphere and the surface of the object. The same algorithm is independently used to generate the surface meshes of the epicardium and endocardium of the four cardiac chambers. The generated meshes are smooth despite the strong voxel anisotropy, which is not the case for the marching cubes and related methods. While the proposed method generates more regular mesh triangles than the marching cubes and allows for a complete control of the number of triangles, the generated meshes are still close to the ones obtained by the marching cubes. The method was tested on 3D short-axis cardiac MR images with strongly anisotropic voxels in the long-axis direction. For the five tested subjects, the average in-slice distance between the meshes generated by the proposed method and by the marching cubes was 0.4 mm.

## 1. Introduction

**Figure 1:**
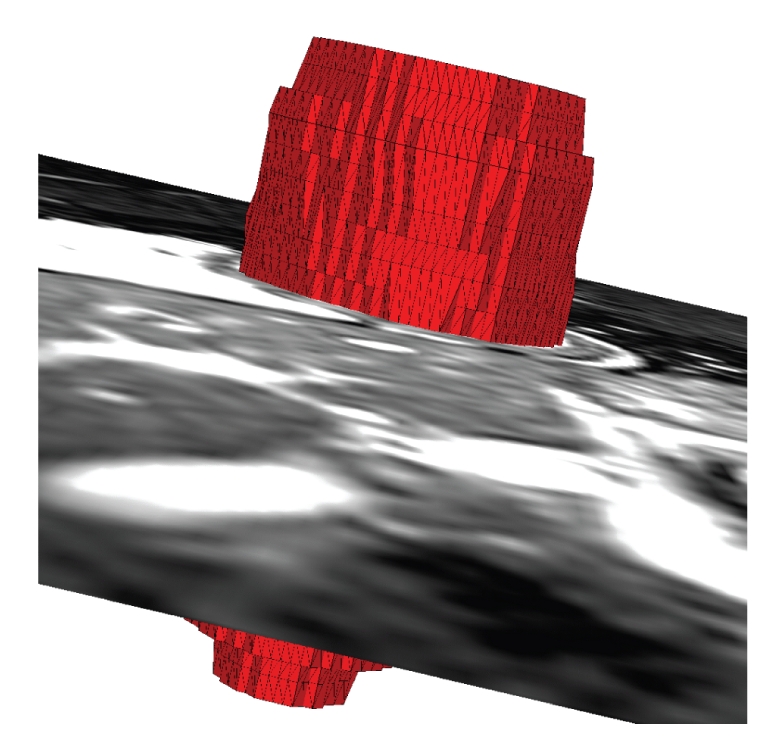
A left ventricular surface model generated by applying the marching cubes algorithm to a segmented cardiac MR image with 1.44 mm in-plane resolution and 8.0 mm slice thickness. The irregular triangles are a consequence of the voxel anisotropy. The surface mesh has pronounced terracing artifacts, and the number of triangles is directly related to the number of voxels in the image.

Surface models of the epicardium and endocardium of the heart chambers are used in a number of biomedical applications for visualization [1], virtual reality [2], segmentation [3, 4], motion analysis [5, 6], shape analysis [7, 8], and modeling [9, 10] purposes. A typical approach to generate subject specific models is to apply a surface construction algorithm to segmented cardiac magnetic resonance (MR) or computed tomography (CT) images. Cardiac MRI is a widely used modality to image the heart. However, due to the tradeoff between image quality and temporal and spatial resolution, voxels in cardiac MR images are strongly anisotropic. Typically, the in-plane resolution is a few times higher than the out-of-plane resolution for clinically used cardiac MRI. While a number of mesh generation methods exist [11, 12], to the best of our knowledge, there is no surface mesh generation method designed for images with strongly anisotropic voxels. The most widely used method for surface mesh generation from images is the marching cubes method [13]. If the marching cubes method is applied to an image with strongly anisotropic voxels without any additional processing, then the generated mesh has strongly irregular triangles, pronounced terracing artifacts and the number of triangles directly related to the number of voxels, none of which is desirable (Figure 1). Terracing artifacts are a consequence of marching cubes mesh closely following sharp voxel boundaries (instead of smoothly fitting them), they appear as “stairs” or “terraces” and in Figure 1 they are visible as dark and light red vertical stripes. Once the marching cubes mesh is generated, one can apply a number of techniques to improve the mesh quality, including mesh smoothing [14], simplification [15], and optimization [16]. A combination of these techniques was used in [17]. An alternative way to use the marching cubes is to first construct an implicit surface from the segmented image, and then apply the marching cubes to the scalar field (the implicit surface is the zero level set of the scalar field) on a uniformly sampled image domain [18]. This way one can control the size of the mesh triangles (determined by the sampling interval) as well as make them more regular (consequence of the uniform sampling), but they can still be badly shaped and the terracing artifacts remain. To alleviate these problems, Peiró et al. [18] used a number of mesh optimization techniques. Instead of using the discrete image, one can interpolate image intensities, for example by means of trilinear interpolation, before constructing a surface mesh [19]. However, in the case of strongly anisotropic voxels, this approach would still result in terracing artifacts, although somewhat smoothed. Lötjönen et al. [20] also used the marching cubes as the starting point of their mesh generation method, which, if the mesh is decimated enough, generates close-to-regular triangles. The method of Gibson [21], while significantly reducing terracing artifacts, shares with the marching cubes the problem of irregular triangles in the case of anisotropic voxels. Another group of methods constructs surface meshes from 2D contours of the segmented image structures [22–25]. They suffer from the same problem: if the voxels of the underlying image are anisotropic the resulting triangles are irregular. We also note related work in which researchers used a structured volumetric mesh of the left ventricle for myocardial motion recovery [26–29].

In this paper we present a method for generation of myocardial wall surface meshes from segmented MRI. The meshes are smooth and have prespecified number of triangles and close-to-regular triangles despite the highly anisotropic voxels. Since the marching cubes are the most widely used method, either as a stand-alone method or as a part of other methods, we compare the proposed method to the marching cubes.

## 2. Methods

### 2.1. Approach

**Figure 2:**
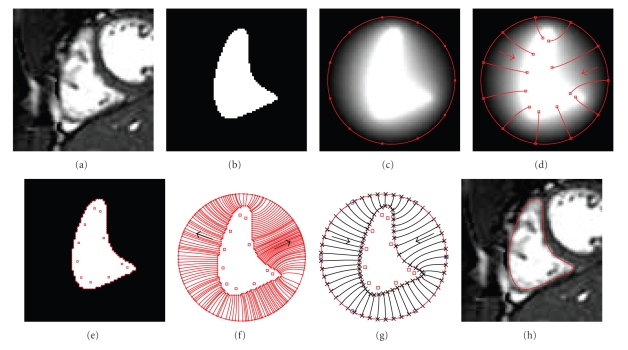
Mesh generation summary. The input image (a) is segmented into the object and background, resulting in a binary image (b). A sphere enclosing the object is centered at the object barycenter (c). The sphere is uniformly sampled with the number of points equal to the number of singularities. The binary image is resampled with isotropic voxels and the Laplace equation is numerically solved between the sphere (boundary condition of 0) and the object (boundary condition of 1). The solution of the Laplace equation is encoded in the gray levels in (c) and (d). The binary object is eroded, and the points are propagated from the sphere to the eroded object in the direction of the gradient of the Laplace equation solution to define the singularity locations, shown as red squares in (d) and (e). Boundary points, specified as midpoints for each pair of neighboring voxel, where one voxel is in the object and the other is in the background, are shown as red dots in (e). The singularity locations as well as the boundary points are used to specify the analytic solution of the Laplace equation. The boundary points are propagated in the negative gradient direction of the solution of the Laplace equation from the object boundary to the sphere (f). Their values of the underlying solution of the Laplace equation are interpolated at the sphere to the define the stopping function. The number of degrees of freedom of the stopping function is defined by the number of control points, which are shown as blue circles in (g). An approximately uniform mesh is generated on the sphere. The vertices of the mesh on the sphere, shown as black crosses in (g), are propagated from the sphere in the direction of the gradient of the solution of the Laplace equation until the value of the underlying solution of the Laplace equation is equal to the corresponding value of the stopping function. The propagated mesh nodes define the final mesh, shown in (h). Figures (a)–(h) are two dimensional for illustration purposes, while the method is three dimensional.

The presented method is designed for surface mesh generation of the endocardium of the four cardiac chambers and of the endocardium from segmented cardiac MRI. The four endocardial surfaces are, if the valves are ignored, topologically equivalent to a sphere. We also assume that the segmentation of the entire heart does not include other structures, which makes its outer surface topologically equivalent to a sphere. The main idea is to generate a triangulated mesh on a sphere and then map it independently to the five surfaces. For each segmented object we construct its surface in implicit form and then map the mesh from the sphere to the surface using the gradient field of the solution of the Laplace equation between the surface and the sphere. Each step of the method is explained in the following sections, and Figure 2 summarizes the method.

### 2.2. Sphere Triangulation

It can be shown that a sphere cannot be triangulated with an arbitrary number of equilateral triangles. In fact, there are only three configurations of a triangulated sphere with equilateral triangles: regular tetrahedron (4 equilateral triangles), regular octahedron (8 equilateral triangles), and regular icosahedron (20 equilateral triangles) [30]. A triangulation of a sphere with any other number of triangles cannot have all the triangles equilateral. There are a number of ways to approximately uniformly sample a sphere and construct the corresponding triangulation [31, 32]. Here we use the method of minimizing the electrostatic energy of equally charged particles on a sphere [33, 34]. Once the points are approximately uniformly distributed on a sphere, we construct a triangular mesh by using the Delaunay triangulation [35]. This method allows for the construction of a close-to-regular triangular mesh on a sphere with an arbitrary number of vertices *V*, which is related to the number of triangles *T* as 2*V* − *T* = 4. This relationship follows from the Euler's formula for polyhedra [30].

### 2.3. Solution of the Laplace Equation

In order to construct the surface mesh, we define a homeomorphic mapping from the sphere to the surface and apply it to the mesh on the sphere. There are infinitely many ways to construct such a mapping, and here we define a scalar field *u* between the sphere and the surface (we make the sphere larger than the object and center it at the barycenter of the object), and then any point from the sphere is mapped to the surface by following ∇*u*, the gradient of *u*. In order for the mapping to be homeomorphic, the gradient flow has to be divergence free, that is, div ∇*u* = 0, which leads to the familiar Laplace equation

(1) Δu=0.

We look for the solution of the Laplace equation that is equal to 0 at the sphere and 1 at the object surface. The Laplace operator is rotation invariant, and a solution to the Laplace equation has no local extrema, which makes it a suitable means to transport the mesh from the sphere to the surface of the object. If the sphere has a radius of *R*, then the external boundary condition can be specified as

(2) $$u\left(r\right){|}_{\left|r\right|=R}=0.$$

The internal boundary condition is discussed in Section 2.4. We use the method of fundamental solutions to solve the Laplace equation. The solution is continuous (as opposed to discrete) and it is represented as a linear combination of functions, each satisfying the Laplace equation and the external boundary condition and each having a singularity within the sphere (see appendix)

(3) $$u\left(r\right)=\overset{M}{\underset{m=1}{\sum }}c_{m}f_{s_{m}}\left(r\right),$$

where **s**_*m*_, *m* = 1,…, *M*, are the locations of *M* singularities and *c*_*m*_ the corresponding coefficients. It is straightforward to show that *u* from (3) satisfies the Laplace equation (1) and the external boundary condition (2).

### 2.4. Internal Boundary Condition

While expression (3) and the external boundary condition are in the continuous form, the internal boundary is discrete, defined by the object segmentation map. The strongly anisotropic voxels are the main reason for irregular triangles and terracing effects present in the meshes generated by methods based on the marching cubes. For this reason, we represent the internal boundary in the continuous form as a level set of *u* from (3). First, we define the set of boundary points **r**_*n*_, *n* = 1,…, *N*. For each pair of neighboring voxels from the segmentation map that have different labels (i.e., one voxel belongs to the object and one does not) the midpoint between the two voxels is a boundary point. Then, we determine the parameters (singularity locations and coefficient values) of *u* such that its level set of 1 fits the boundary points in the least squares sense, that is, we minimize

(4) $$O=\frac{1}{2}\overset{N}{\underset{n=1}{\sum }}{\left[u\left(r_{n}\right)-1\right]}^{2}.$$

We require all the singularities to be located within the object. Each singularity has the corresponding singularity outside the sphere (see the appendix). Scalar field *u* is well defined in the domain (between the object surface and the sphere) since there are no singularities. However, there is no closed form solution that involves the optimal locations of the singularities. To avoid numerical optimization which is prone to local extrema, we preset the singularity locations, and then find the optimal coefficients *c*_*m*_ that minimize (4). While a closed form solution for the optimal coefficients can easily be obtained, in the general case some of the optimal coefficients can have positive and some negative values. However, around each singularity with a negative coefficient there will be a region with negative values of *u* (*u* will tend to −∞ at such singularities). These “islands” of negative values may be fully contained within the object but they also may protrude into the domain between the object surface and the sphere, affecting the 1 level set in an undesired way. To prevent this from happening, one can constrain the optimization to have only positive values for *c*_*m*_. However, there is no closed form solution of this problem. To avoid numerical optimization which is prone to local extrema, we resort to an alternative approach. We approximately uniformly place the singularities inside the object relatively close to the object surface (see Section 2.6), and assume that all the coefficients have the same value, that is, *c*_*m*_ = *c*. The optimal value of *c* is obtained from

(5) $$\frac{\partial O}{\partial c}=0,$$

which has a closed form solution

(6) $$c=\frac{\sum_{n=1}^{N}d_{n}}{\sum_{n=1}^{N}d_{n}^{2}},$$

where *d*_*n*_ = ∑_*m* = 1_^*M*^ *f*_*m*_(**r**_*n*_). It can be shown that *c* > 0, that is, there will be no singularities with negative coefficients, which could lead to undesired mesh shapes. However, since the same coefficient is used for all the singularities, the fitting of the implicit surface (level set 1 of *u*) to the boundary points is not as accurate as in the case of singularities with nonequal coefficients. To increase the accuracy of the fitting, we use a stopping function (see Section 2.7).

### 2.5. Mesh Propagation from the Sphere to the Surface

To map the mesh from the sphere to the object surface, we propagate each mesh vertex along the gradient of *u*; that is, the trajectory **r**(*t*) of a given vertex **v** on the sphere is

(7) $$\begin{array}{cc} \frac{dr\left(t\right)}{dt} & =\nabla u\left(r\left(t\right)\right),\\ r\left(0\right) & =v, \end{array}$$

where *t* is the parameter of the trajectory. We use the fourth-order Runge-Kutta method [36] to integrate the trajectory numerically. Since *u* is a continuous and exact solution of the Laplace equation, the only propagation error comes from the numerical integration error of the Runge-Kutta method. If the Laplace equation was solved approximately, the nonexactness of the solution would be translated into additional propagation error.

### 2.6. Placement of Singularities

To uniformly place the singularities inside the object relatively close to the object surface, we first approximately uniformly sample the sphere (as explained in Section 2.2) with the number of points equal to the number of singularities. Then, we resample the segmentation map to obtain isotropic voxels and erode the segmented object two times. In the next step, we numerically solve the Laplace equation between the sphere (with a boundary condition of 0) and the surface of the eroded object (with a boundary condition of 1) by using a relaxation method [36]. We utilize the isotropic voxels as the grid on which we solve the Laplace equation. Finally, we propagate the points from the sphere to the eroded object in the direction of the gradient of the solution of the Laplace equation to obtain the singularity locations.

### 2.7. Stopping Function

To increase the accuracy of the fitting of the object surface to the boundary points, instead of propagating the mesh from the sphere to the level set of 1, we define a “stopping” function on the sphere, which for every point on the sphere determines the value of *u* that point will be propagated to (that value would be 1 if the mesh was propagated to the level set of 1).

To represent the stopping function we use pseudothin plate spline model on the sphere proposed by Wahba [37]

(8) $$b\left(\hat{p}\right)=\alpha_{0}+\overset{K}{\underset{k=1}{\sum }}\alpha_{k}\psi \left(\hat{p}\cdot {\hat{q}}_{k}\right),$$

where $\hat{p}$ is a unit vector representing a point on the sphere, *K* is the number of control points, ${\hat{q}}_{k}$ are unit vectors defining the control points on the sphere, *α*_0_,…, *α*_*K*_ are scalar coefficients, and *ψ* is defined in [37] for *m* = 4. We set the control points ${\hat{q}}_{k}$ by approximately uniformly sampling a sphere (as explained in Section 2.2) with *K* points.

At this point the singularity locations as well as coefficient *c* are set; that is, the scalar field *u* is completely defined. At each boundary point **r**_*n*_ we record the value of the scalar field *u*(**r**_*n*_). Let ${\hat{p}}_{n}$ denote the unit vector of the point on the sphere obtained by propagating the boundary point **r**_**n**_ to the sphere by following the negative gradient of *u*. We determine *α*_0_,…, *α*_*K*_ by requiring $b\left(\hat{p}\right)$ at points ${\hat{p}}_{n}$ to approximate values *u*(**r**_*n*_) in the least squares sense, that is, by minimizing

(9) $$\begin{array}{c} \overset{N}{\underset{n=1}{\sum }}{\left[b\left({\hat{p}}_{n}\right)-u\left(r_{n}\right)\right]}^{2}. \end{array}$$

The closed form solution is

(10) $$\begin{array}{c} \left[\begin{array}{c} \alpha_{0}\\ \alpha_{1}\\ \vdots \\ \alpha_{k} \end{array}\right]={\left(G^{T}G\right)}^{-1}G^{T}\;\left[\begin{array}{c} u\left(r_{1}\right)\\ u\left(r_{2}\right)\\ \vdots \\ u\left(r_{N}\right) \end{array}\right], \end{array}$$

where

(11) $$\begin{array}{c} G=\left[\begin{array}{cccc} 1 & \psi \left({\hat{p}}_{1}\cdot {\hat{q}}_{1}\right) & \cdots & \psi \left({\hat{p}}_{1}\cdot {\hat{q}}_{K}\right)\\ 1 & \psi \left({\hat{p}}_{2}\cdot {\hat{q}}_{1}\right) & \cdots & \psi \left({\hat{p}}_{2}\cdot {\hat{q}}_{K}\right)\\ \vdots & \vdots & & \vdots \\ 1 & \psi \left({\hat{p}}_{N}\cdot {\hat{q}}_{1}\right) & \cdots & \psi \left({\hat{p}}_{N}\cdot {\hat{q}}_{K}\right) \end{array}\right]. \end{array}$$

Coefficient *c* is computed such that (4) is minimized, which means that the values *u*(**r**_*n*_) are close to 1. Coefficients *α*_0_,…, *α*_*K*_ are computed such that (9) is minimized, which means that $b\left({\hat{p}}_{n}\right)$ is close to *u*(**r**_*n*_), which in turn is close to 1. Since typically points ${\hat{p}}_{n}$ relatively densely cover the sphere, the stopping function $b\left(\hat{p}\right)$ is close to 1 everywhere on the sphere.

We propagate the mesh vertices from the sphere to the object surface according to (7). For a given mesh vertex **v** on the sphere, the corresponding unit vector is **v**/|**v**| and the value of the stopping function for that vertex is *b*(**v**/|**v**|). Over the course of propagation the underlying value of the scalar field *u*(**r**(*t*)) grows and when it reaches *b*(**v**/|**v**|) the propagation stops.

Instead of using the value of 1 to stop all the vertices, we increase the accuracy of the fitting of the boundary points by using the stopping function. The more control points the more accurate the fitting, and the fewer control points the smoother the final surface.

### 2.8. In-Slice Distance Calculation

To quantify the closeness of the meshes generated by the marching cubes and the proposed method we compute the in-slice distance between the respective mesh cross-sections in a given slice (results given in Section 3.4). To measure the in-slice distance we densely sample the two cross-sections. For a given point (**p**_1_) in the first set of samples we find the closest point (**p**_2_) in the second set of samples and then for **p**_2_ we find the closest point (**p**_3_) in the first set of samples. If **p**_3_ is the same as **p**_1_ then we say that **p**_1_ and **p**_2_ form a pair of corresponding points in the two cross-sections. We find all the pairs of corresponding points in the two cross-sections and compute their distances, from which we compute the average distance and standard deviation.

## 3. Results

### 3.1. Test Images

The method was tested using anatomical cardiac MRI scans of five healthy volunteers. The scans were acquired using steady-state free-precession short axis cine imaging with a 1.5 T clinical MRI scanner (Intera, Philips Medical Systems, Best, The Netherlands). The scans had 12-17 contiguous short axis slices with 256 × 256 pixels, 8 mm slice thickness, 1.44 mm in-plane resolution and a 20 cm field of view. A flip angle of 65°, TR of 3.4  milliseconds, and TE of 1.7 milliseconds were used. The four cardiac chambers as well as the entire heart were manually segmented in all five scans.

### 3.2. Method Parameters

The presented method has three parameters: the number of singularities (*M*), the number of control points (*K*), and the number of mesh vertices (*V*). Note that *M* and *K* affect the shape of the continuous implicit surface, while *V* affects the triangulation of the continuous implicit surface.

In this section we analyze the effect of the parameter values on the resulting mesh. The studies were done on the right ventricle of one of the subjects, since the right ventricle is more curved than the other three chambers and is the only chamber that has both convex and concave regions.

**Figure 3:**
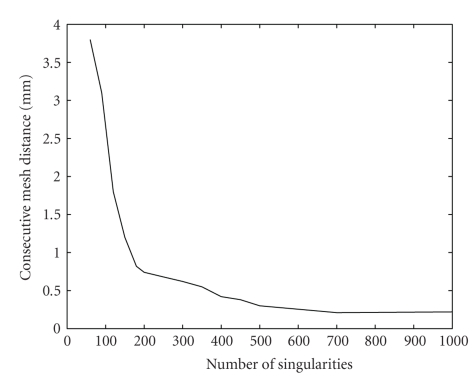
Average distance between consecutive meshes as a function of the number of singularities.

In the first study we generated a sequence of surface meshes by increasing the number of singularities. Then we computed the average distance between consecutive meshes in the sequence to quantify the change the mesh undergoes as *M* is increased (Figure 3). 

**Figure 4:**
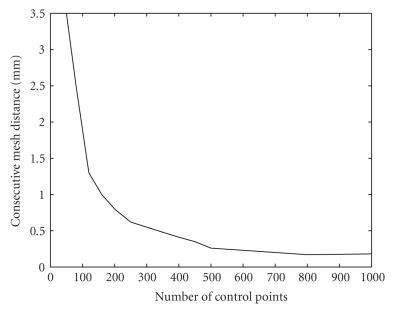
Average distance between consecutive meshes as a function of the number of control points.

 In the second study we generated a sequence of surface meshes by increasing the number of control points. Then we computed the average distance between consecutive meshes in the sequence to quantify the change the mesh undergoes as *K* is increased (Figure 4). 

**Figure 5:**
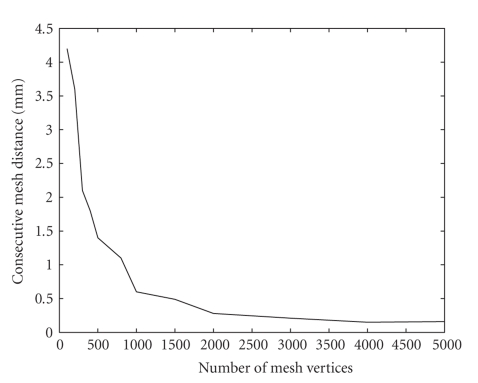
Average distance between consecutive meshes as a function of the number of mesh vertices.

In the third study we generated a sequence of surface meshes by increasing the number of mesh vertices. Then we computed the average distance between consecutive meshes in the sequence to quantify the change the mesh undergoes as *V* is increased (Figure 5). 

### 3.3. Mesh Quality

To measure the mesh quality, we use a triangle quality index suggested in [18], which can be evaluated as

(12) $$Q=\frac{8\left(p-a\right)\left(p-b\right)\left(p-c\right)}{abc},\;p=\frac{a+b+c}{3},$$

where *a*, *b*, and *c* are the lengths of the three sides of the triangle. It can be shown that 0 ≤ *Q* ≤ 1 for any triangle, *Q* = 1 for an equilateral triangle, *Q* is close to zero for irregular triangles, and *Q* = 0 for a zero-area triangle. Triangles with *Q* > .5 are considered to be of reasonably good quality.

**Figure 6:**
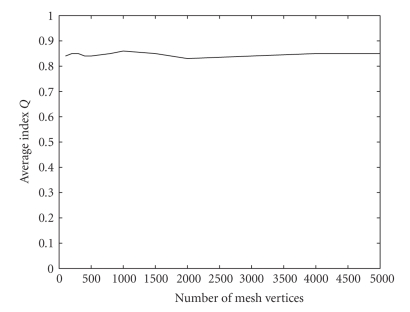
Average triangle quality index as a function of the number of mesh vertices.

We generated a sequence of surface meshes by increasing the number of mesh vertices. Then we computed the average quality index for each mesh in the sequence (Figure 6). One can see that the quality index is practically independent of the number of mesh vertices and that it is relatively high (*Q* ≈ .85).

**Figure 7:**
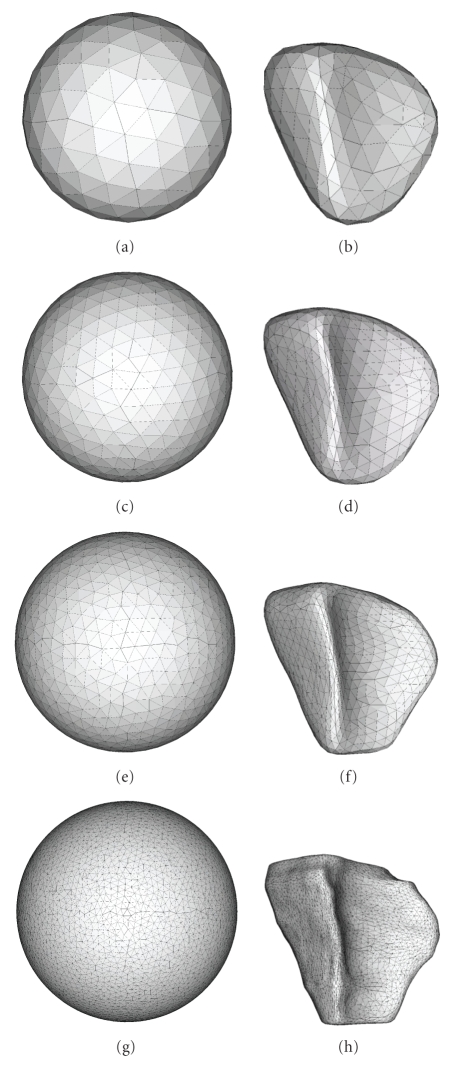
Each row shows a mesh on the sphere and the corresponding right ventricular mesh obtained by propagating the mesh from the sphere to the right ventricular surface. The numbers of mesh vertices for the four rows are 200, 500, 1000, and 5000. The corresponding mean ± std (min, max) *Q* values for the mesh on the sphere are 0.93 ± 0.07(0.75, 1), 0.94 ± 0.06(0.78, 1), 0.93 ± 0.07(0.77, 1), and 0.95 ± 0.05(0.76, 1), and for the right ventricular mesh are 0.85 ± 0.07(0.68, 0.99), 0.84 ± 0.06(0.62, 0.99), 0.86 ± 0.06(0.65, 0.99), and 0.85 ± 0.07(0.63, 0.99).

It should be noted that the average *Q* cannot be 1. This is true even for the sphere, since it cannot be triangulated with an arbitrary number of equilateral triangles. Figure 7 shows the mesh on the sphere and the corresponding right ventricular mesh for four different numbers of mesh vertices and reports the corresponding *Q* values. 

### 3.4. Comparison to the Marching Cubes

**Figure 8:**
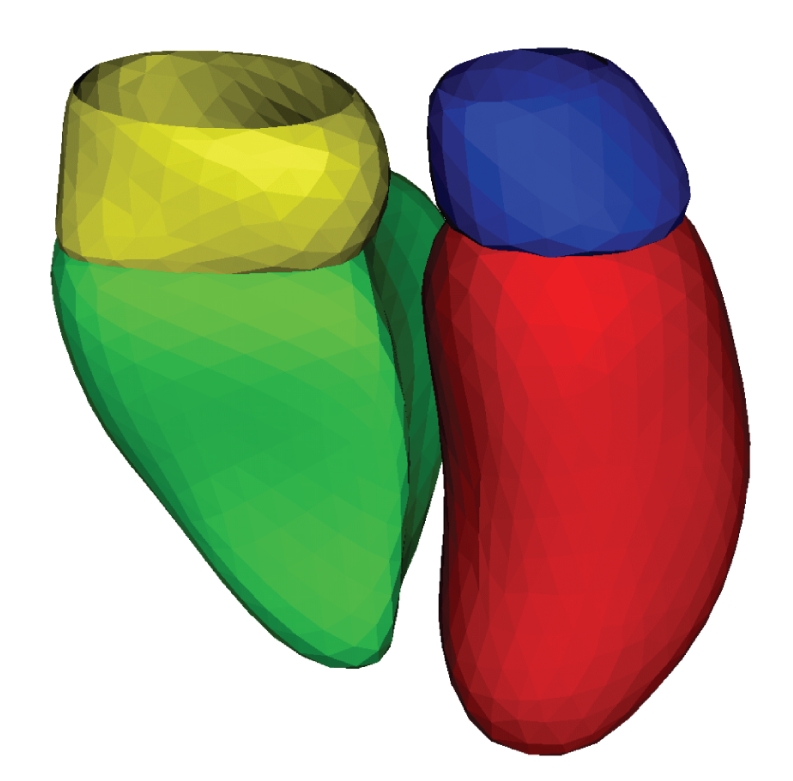
Endocardium surface meshes generated by the proposed method for the left ventricle (red), right ventricle (green), left atrium (blue), and right atrium (yellow).

**Figure 9:**
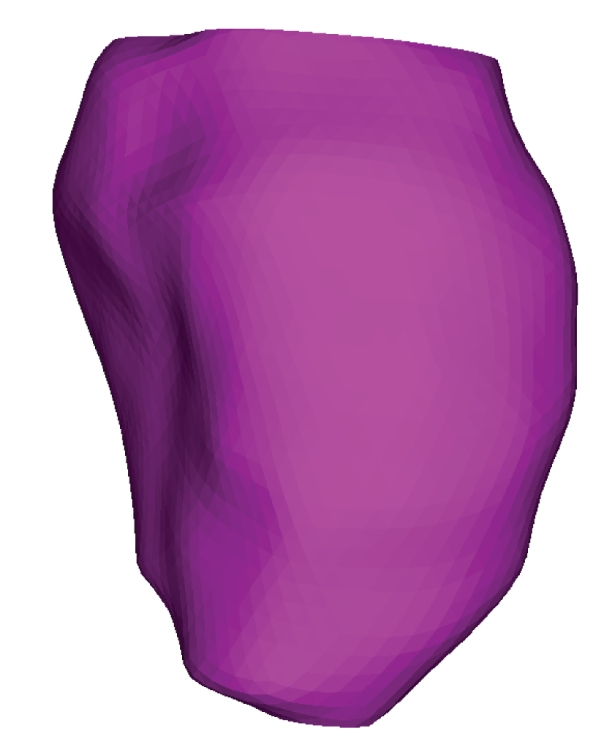
Epicardium surface mesh generated by the proposed method for the entire myocardium.

The method was tested on the endocardial surfaces of the four cardiac chambers as well as on the epicardial surface of the entire heart for five subjects. The numbers of singularities, control points, and mesh vertices used for the test are reported in Table 1. Figures 8 and 9 show the endocardial and epicardial surface meshes for one of the subjects. 

**Figure 10:**
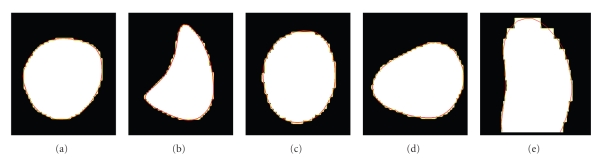
Contours of endocardial meshes generated by the marching cubes (yellow) and the proposed method (red) in short-axis sections for (a) left ventricle, (b) right ventricle, (c) left atrium, and (d) right atrium, and in a long-axis section for (e) left ventricle. The endocardial boundaries are defined by the blood pool segmentation shown in the binary images.

Since the marching cubes are the most widely used method, either as a stand-alone method or as a part of other methods, we compared the endocardial and epicardial surface meshes of the five subjects generated by the proposed method to the corresponding surface meshes generated by the marching cubes. The comparison was done in the short-axis slices since the in-plane resolution was 5 times higher than the out-of-plane resolution. Figure 10 shows cross-sections of segmented blood pools and the corresponding contours from endocardial meshes obtained by the marching cubes and the proposed method. Table 1 provides the average in-slice distance between the surface meshes generated by the marching cubes and the proposed method. The averages were computed over all the short-axis slices of each cardiac chamber for the five subjects. 

## 4. Discussion

 The presented method can be used for the surface mesh generation of any object that is topologically equivalent to a sphere. While the method can be extended to control the triangle size based on the surface curvature, there is no need for such an approach in the case of myocardial wall surfaces since they are not highly curved. The method, unlike other mesh generation methods, allows for a direct control of the number of triangles and vertices in the mesh, which is particularly useful in modeling (e.g., FEM) applications.

The method can be used for the generation of surfaces that are not closed. For example acquired cardiac MRI might not contain slices going through the apex and the base, in such case the corresponding endocardial and epicardial surfaces are not closed. In such cases one can segment the acquired slices, generate the mesh using the proposed method and then clip the bottom and top part of the mesh. This was done for the two atria in Figure 8 and for the epicardium in Figure 9.

In the examples presented in this paper we generated triangulated surface meshes. However, the method is independent of the type of the mesh; that is, it can be used with any mesh elements as long as the sphere can be meshed with such elements. Once the sphere is meshed, the vertices of the mesh are propagated from the sphere to the surface of the segmented object in the way explained in Section 2.5.

Figures 3 and 4 show that the method converges as the number of singularities or the number of control points is increased, which is a desirable behaviour. It means that beyond certain number of singularities and control points the method behaves the same. By comparing Figures 3 and 4 one can see that the two parameters have very similar behaviour, and this is why we used similar values for the two parameters (Table 1) when we compared our method to the marching cubes (Section 3.4). The two parameters have the same effect: they control the smoothness of the underlying continuous surface. The higher their values the smoother the surface, and the lower their values the better the surface fits the boundary points. For these reasons, one can use the same value for the two parameters and treat them as one parameter.

While the number of singularities and the number of control points control the smoothness of the underlying implicit continuous surface, the number of mesh vertices affects the triangulation of the continuous surface. From Figure 5 one can see that the method converges as the number of vertices is increased, which is a desirable behaviour.

The reason why the graphs in Figures 3, 4, and 5 do not exactly go to zero is the numerical errors in the implementation of the method resulting in submillimeter differences in the final location of the mesh vertices. We use a continuous and exact solution of the Laplace equation, since an approximate solution would increase the numerical errors.

The constant and relatively high value of the triangle quality index in Figure 6 shows that the mesh has close-to-regular triangles for a range of numbers of vertices. The same conclusion can be made from Figure 7.

The proposed method generates meshes that are very close to the ones obtained by the marching cubes (Figure 10). While the differences in the short axis planes between the meshes generated by the two methods were submillimeter (Table 1), the meshes generated by the proposed method had about five times fewer triangles than the corresponding meshes generated by the marching cubes. However, unlike the meshes generated by the marching cubes (Figure 1), the meshes generated by the proposed method (Figures 8 and 9) are smooth and have close-to-regular triangles.

The proposed method can be used with segmented images that have anisotropic voxels. The segmentation boundary points are not strictly interpolated. Instead, they are approximated with an implicit surface that fits them in the least square sense. The surface smoothness versus the goodness of fit is controlled by the number of singularities and number of control points, which can take the same value. If the implicit surface is smooth then the resulting mesh is also smooth. Thus, there is no need for artificial smoothing of the mesh that may shrink or affect the mesh in some other undesired way.

The entire method has been designed to completely avoid numerical optimization and consequently the problem of local extrema.

In the proposed method the segmentation boundary points are approximated with an implicit surface, which is then triangulated by propagating a regular mesh from a sphere to the surface. There are other ways to construct continuous surfaces that interpolate or approximate a given set of points (e.g., [38]). However, our representation allows for an exact continuous solution of the Laplace equation, while other surface models would require a numerical solution, which in turn would increase the mesh propagation error. Instead of propagating a regular mesh from the sphere to the surface, one can use a method for direct triangulation of implicit surfaces (e.g., [39–41]). These methods are more general than our method since they can deal with an arbitrary topology. They march triangles over the surface and use heuristics to close the triangulation. Unlike these methods, the proposed method, while restricted only to spherical topology, does not need a heuristics to close the triangulation and it can generate meshes with a prespecified number of triangles.

We note that harmonic functions have already been used to represent shapes [42] and that there are other ways to map a sphere to the surface or vice versa (e.g., [43]).

In the myocardial motion analysis community researchers used a structured volumetric mesh of the left ventricle [26–29]. The model was defined in the prolate spheroidal coordinate system, and its epicardial and endocardial surfaces were fit to left ventricular wall boundary points extracted from cardiac MRI. The obtained continuous smooth model was meshed by mapping a premeshed ellipsoid to the model. A disadvantage of this approach is that the size and regularity of the mesh elements is not uniform; rather, it depends on the distance of the mesh elements from the pole of the prolate spheroidal coordinate system. Our proposed approach is similar to theirs in that we also fit a continuous smooth model to boundary points extracted from images and then map a premeshed sphere to the model. However, our model does not have poles or any other special points and all the mesh elements are uniform in size and regularity.

## 5. Conclusion

We have developed a novel method for the construction of endocardial and epicardial surface meshes from 3D segmented cardiac MR images with a prespecified number of vertices and triangles. Even when the voxels are strongly anisotropic, the resulting meshes are smooth and have close-to-regular triangles while closely following the segmentation.

## Figures and Tables

**Table 1:** The average in-slice distance (*D*) between the surface meshes generated by the marching cubes and the proposed method is given for each mesh. The in-plane resolution was 1.44 mm × 1.44 mm. The numbers of singularities (*M*), control points (*K*), and mesh vertices (*V*) used to generate the meshes with the proposed method are also reported.

	*M *	*K *	*V *	*D* [mm]
LV endocardium	227	204	1180	0.4 ± .07
RV endocardium	246	225	1220	0.3 ± .05
LA endocardium	62	66	748	0.5 ± .06
RA endocardium	57	58	684	0.4 ± .05
Epicardium	422	406	2472	0.3 ± .05

## Appendix

### Harmonic Function with a Spherical Isolevel and Single Singularity

This section describes the solution to the Laplace equation over a spherical domain that has a single singularity somewhere within the domain and that is equal to a constant on the boundary of the domain. Let the sphere center be the coordinate system origin, *R* denote the radius of the sphere, **s** the location of the singularity, and **r** the independent variable. The solution *f*_**s**_(**r**) needs to satisfy the following:

(A.1) $$\begin{array}{c} \underset{r\to s}{\lim\;}f_{s}\left(r\right)=\infty , \end{array}$$

(A.2) $$\begin{array}{c} f_{s}\left(r\right){|}_{\left|r\right|=R}=0, \end{array}$$

(A.3) $$\begin{array}{c} \Delta f_{s}=0. \end{array}$$

The fundamental solution of the Laplace equation in 3D is 1/|**r**| and it represents a singularity at the origin. The fundamental solution centered at **s**, that is, function 1/|**r** − **s**|, satisfies (A.1) and (A.3), but it does not satisfy the boundary condition (A.2). It turns out that the sum of two shifted fundamental solutions, one centered at **s** and one centered at **s** *R* ^2^/|**s**|^2^ and multiplied by −*R*/|**s**|, satisfies (A.1), (A.2), and (A.3). Note that the second singularity is outside the sphere, that is, there is only one singularity within the spherical domain. The solution is

(A.4) $$\begin{array}{c} f_{s}\left(r\right)=\frac{1}{\left|r-s\right|}-\frac{R}{\left|s\right|}\frac{1}{\left|r-sR^{2}/{\left|s\right|}^{2}\right|}, \end{array}$$

or, alternatively

(A.5) $$\begin{array}{c} f_{s}\left(r\right)=\frac{1}{\sqrt{{\left|r\right|}^{2}-2rs+{\left|s\right|}^{2}}}-\frac{R}{\sqrt{{\left|s\right|}^{2}{\left|r\right|}^{2}-2R^{2}rs+R^{4}}}. \end{array}$$

It is straightforward to show that (A.5) satisfies (A.1), (A.2), and (A.3). Also, *f*_**s**_(**r**) > 0 when |**r**| < *R*, *f*_**s**_(**r**) = 0 when |**r**| = *R*, and *f*_**s**_(**r**) < 0 when |**r**| > *R*. In (A.2) it is assumed that *f*_**s**_ is zero at the domain boundary. The zero can be replaced by a constant *C* by simply adding *C* to *f*_**s**_. The gradient of *f*_**s**_ is

(A.6) $$\begin{array}{cc} \nabla f_{s} & =\frac{\partial f_{s}}{\partial r}\\ & =\frac{s-r}{{\left({\left|r\right|}^{2}-2rs+{\left|s\right|}^{2}\right)}^{3/2}}+\frac{R\left({\left|s\right|}^{2}r-R^{2}s\right)}{{\left({\left|s\right|}^{2}{\left|r\right|}^{2}-2R^{2}rs+R^{4}\right)}^{3/2}}. \end{array}$$

Expression (A.6) is used in the evaluation of ∇*u* in (7).
